# Cross-cultural adaptation, reliability and validity tests of the Chinese version of the Profile Fitness Mapping neck questionnaire

**DOI:** 10.1186/s12891-022-06087-x

**Published:** 2023-01-12

**Authors:** Lu Qi, Rui Chang, Enming Zhang

**Affiliations:** School of Sports Medicine and Rehabilitation, Beijing Sport University, No.48 Xinxi Road, Haidian District, Beijing, 100084 China

**Keywords:** Chronic neck pain, The Chinese version of ProFitMap-neck, Cross-cultural adaptation, Reliability and validity tests

## Abstract

**Objective:**

To translate and culturally adapt the Profile Fitness Mapping neck questionnaire (ProFitMap-neck) into the Chinese version and evaluate its psychometric properties.

**Methods:**

The procedure of translation and cross-cultural adaptation was performed according to the recommended guidelines. A total of 220 patients with chronic neck pain (CNP) and 100 individuals without neck pain participated in the study. Internal consistency, test-retest reliability, content validity and construct validity were investigated.

**Results:**

The Chinese version of ProFitMap-neck (CHN-ProFitMap-neck) showed adequate internal consistency (Cronbach’s α = 0.88–0.95). A good test-retest reliability was proven by the intraclass correlation coefficient (ICC_3A,1_ = 0.78–0.86). Floor-ceiling effects were absent. Exploratory factor analysis revealed 6 factors for the symptom scale and 4 factors for the function scale. The CHN-ProFitMap-neck showed a moderate to high negative correlation with NDI (*r* = 0.46–0.60, *P* < 0.01), a small to moderate negative correlation with VAS (*r* = 0.29–0.36, *P* < 0.01), and a small to high positive correlation with SF-36 (*r* = 0.21–0.52, *P* < 0.01). No significant correlation between the CHN-ProFitMap-neck function scale and VAS (*P* > 0.05) or the mental health domain of the SF-36 was found (*P* > 0.05). The CHN-ProFitMap-neck scores were significantly lower in the CNP group than in the non-CNP group (*P* < 0.01).

**Conclusions:**

The CHN-ProFitMap-neck had acceptable psychometric properties and could be used as a reliable and valid instrument in the assessment of patients with chronic neck pain in mainland China.

**Supplementary Information:**

The online version contains supplementary material available at 10.1186/s12891-022-06087-x.

## Introduction

Neck pain leads to pain and functional disability with a negative impact on patients’ function, activity and quality of life [1]. Absence from work due to neck pain causes a heavy burden for individuals, families, and society [2, 3]. 50% ~ 85% general population who once experienced neck pain report recurrent episodes of neck pain within 1 to 5 years [4].

The risk factors of neck pain involve the individual ones (such as gender, age, history of neck pain, low back pain, psychological distress, lack of social support, complications, physical activity, living quality, and education level) [2, 3, 5–7] and the occupational ones (such as sedentary lifestyle, long working period, intense workload, and improper ergonomic device) [2, 8–10]. Due to the multifactorial risk factors and uncertain pathophysiological mechanisms, the assessment of patients with neck pain plays a vital role in formulating treatment plans [11]. The patient-reported outcome measures (PROMs) have become a convenient and efficient tool to assess musculoskeletal problems from the perspectives of patients [12]. PROMs help physicians identify a patient’s baseline status, monitor pain-related changes, function, disability and psychosocial functioning [3], help formulate treatment plans, evaluate treatment effects [11, 13, 14], and facilitate patient involvement in developing treatment plans [15].

The ProFitMap-neck was developed between 1992 and 1994 at the Alfa Rehab Center in Sweden [16]. It consists of a symptom scale (26 items) and a functional limitation scale (18 items); the symptom scale involves a frequency index and an intensity index [16]. Thus, changes in different dimensions (symptom-frequency, symptom-intensity and functional limitation) can be detected separately. Furthermore, the patients’ experience, the pain-related literature, and the experts’ views were considered in the development of the ProFitMap-neck [16], which made its content more comprehensive. It involves factors such as pain characteristics, body distributions, temporal aspects and different senses, which were suggested to be evaluated in pain assessment [17, 18]. Additionally, the ProFitMap-neck considers important dimensions of neck pain according to the International Classification of Functioning, Disability and Health (ICF) categories. The items in the symptom scale could be classified into impairment and environmental factors; the items in the functional limitation scale could be divided into activity limitation and participation restriction [16].

Given the ever-increasing trend for comprehensive assessment of CNP, it is essential to culturally adapt the ProFitMap-neck to further clinical practice. To our best knowledge, the ProFitMap-neck was only translated and culturally adapted into Brazilian Portuguese [19] and Turkish [20]. This study aimed to translate and adapt the ProFitMap-neck into the Chinese version (CHN-ProFitMap-neck) and to investigate its psychometric properties by reliability and validity tests.

## Materials and methods

### Study design

This study was carried out in several universities and companies in Beijing, China and was approved by the Ethics Committee of Beijing Sport University (approval number: 2021070H). The translation and cross-cultural adaptation were conducted according to the procedures established by Beaton et al. [21] After signing written informed consent, all participants independently completed the CHN-ProFitMap-neck. The subjects in the chronic neck pain (CNP) group completed the CHN-ProFitMap-neck, the VAS, the NDI, the SF-36, and demographic variables (such as age, gender and education); subjects in the non-CNP group completed the CHN-ProFitMap-neck. After 1 week, 100 subjects in the CNP group were randomly chosen to answer the CHN-ProFitMap-neck again at the same time as the first test.

### Translation and cultural adaptation

**Fig. 1:**
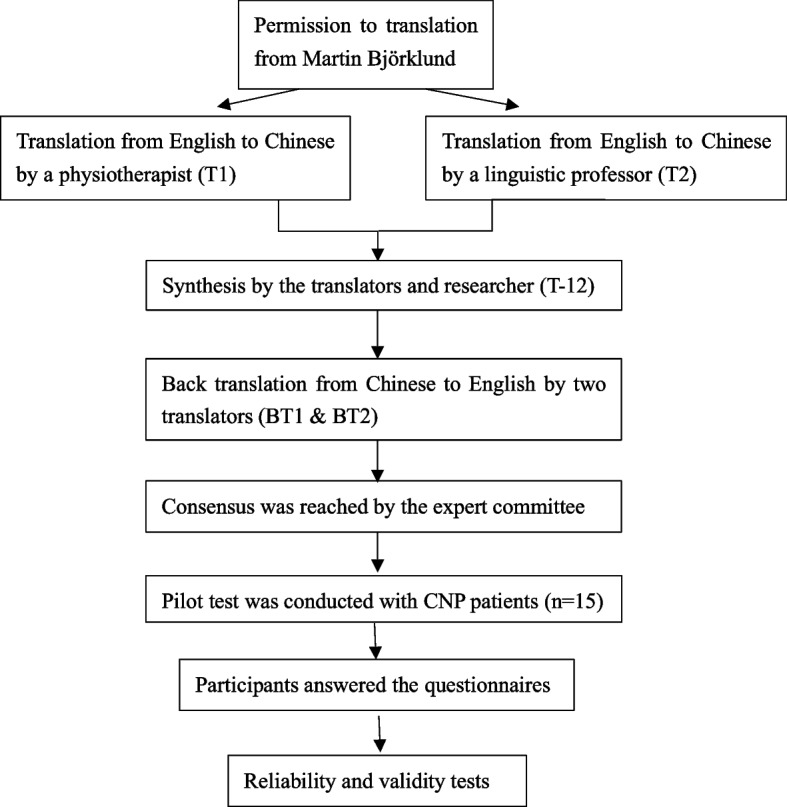
Flowchart of the cross-cultural adaptation, reliability and validity tests of the Chinese version of ProFitMap-neck (CHN-ProFitMap-neck)

First, we contacted the ProFitMap-neck developer, associate professor Martin Björklund, via e-mail to obtain permission for translation and cross-cultural adaptation. Procedures established by Beaton et al [21] were strictly followed (Fig. 1).

#### Stage I: forward translation (English to Chinese)

The English version of the ProFitMap-neck was translated into Chinese by two native Chinese speakers, bilingual with English as a second language (Fig. 1). Translator 1 is a physiotherapist and translator 2 is an English linguistic professor. The two Chinese versions of ProFitMap-neck were named Translation 1 (T1) and Translation 2 (T2), respectively.

#### Stage II: synthesis

A synthesis of the two translations (T1 and T2) was obtained with the discrepancies fully discussed and solved by the translators and researchers of this study (Fig. 1). This process was documented and the synthesis version was named Translation 12 (T-12).

#### Stage III: back translation (Chinese to English)

Two native English speakers with Chinese as a second language translated the T-12 into English (Fig. 1). They have no background in medicine. These two English versions were named Back Translation 1 (BT1) and Back Translation 2 (BT2).

#### Stage IV: expert committee

The expert committee consisted of one methodologist, one health professional, one language professional, and all translators. They reviewed the original and all the translation versions and a prefinal Chinese version of the CHN-ProFitMap-neck was produced (Fig. 1).

#### Stage V: pilot test

Fifteen CNP patients were recruited. All participants completed the CHN-ProFitMap-neck and were interviewed about their comprehensibility of the items, answers, and instructions of the CHN-ProFitMap-neck (Fig. 1). Any problem that raised in this process was also documented and addressed.

Stages I to V could be repeated until no query existed.

### Participants

From September to December 2021, participants were recruited from Beijing, China, via posters and social media. Individuals aged 18 to 65 years old who have the capability of communication, reading and writing were included. The CNP patients experience neck pain for more than 3 months. The exclusion criteria included: (1) experiencing acute neck pain, (2) history of cervical fractures or surgery, (3) vestibular neurological or cardiovascular disease, (4) during pregnancy. The sample should be 4 to 10 times the number of items with a minimum sample size of 100 [22]. In this study, the sample size was 5 times the number of items. Thus, 220 CNP patients and 100 subjects without neck pain were recruited.

### Instruments

#### Profile Fitness Mapping neck questionnaire (ProFitMap-neck)

The ProFitMap-neck consists of a symptom scale (26 items) and a functional limitation scale (18 items). The symptom scale involves a frequency index and an intensity index. The score in the symptom-frequency index ranges from 1 (never/ very seldom) to 6 (very often/ always). The score in the symptom-intensity index ranges from 7 (nothing/ none at all) to 12 (almost unbearable/ unbearable, all/ maximally). The score in the functional limitation scale ranges from 1 (very good, no problem, very satisfying, very likely) to 6 (very bad, very difficult/ impossible, very dissatisfying, very unlikely). The calculation of scores could be seen in the original ProFitMap-neck study [16]. The ProFitMap-neck has proved to be an instrument with good reliability and validity in the assessment of neck pain [16, 19, 20].

#### Visual analogue scale (VAS)

The VAS is a self-reported scale. It consists of a horizontal or vertical line (usually 100 mm) anchored by two verbal phrases at both ends to describe the pain status. One end implies “no pain”, and the other end implies “unbearable pain” [23, 24].

#### Neck disability index (NDI)

The NDI is one of the most widely used self-reported questionnaires to assess neck dysfunction [25]. The NDI has 10 items, including pain severity, personal care, lifting, reading, headache, concentration, working, driving, sleeping and leisure activities [26]. The score of each item ranges from 0 (no pain or dysfunction) to 5 (maximal pain or dysfunction) [25]. The Chinese version of NDI has proven to be a reliable, valid and responsive instrument to measure functional limitations in patients with neck pain [27].

#### The 36-item short form health survey (SF-36)

The SF-36 is a self-evaluated tool consisting of 36 items that can be divided into 8 dimensions: physical functioning (PF), role limitations due to physical problems (RP), body pain (BP), general health (GH), vitality (VT), social functioning (SF), role limitations due to emotional problems (RE), mental health (MH) and one single item of health transition [28]. Each item can be transformed into scores of 0 to 100, and higher scores indicate better function and health status [29]. The Chinese version of the SF-36 was proven to have acceptable psychometric properties [28].

### Statistical analyses

The statistical analyses were carried out using Statistical Package for the Social Sciences (SPSS) Version 23.0 (IBM Corp., Armonk, NY). The demographic and clinical data of the participants were described by means and standard deviations. *P* < 0.05 was considered significant in all statistical tests.

#### Measures of reliability

Internal consistency is the ability of an instrument to involve interrelated items [30]. The internal consistency could be assessed by Cronbach’s α. If Cronbach’s α value is no less than 0.7, the internal consistency is considered adequate [31]. The test-retest reliability indicates the ability of the scores of an instrument to be reproducible over time when it is used on the same patient, whose condition has not changed [30]. The test-retest reliability could be assessed by the intraclass correlation coefficient (ICC_3A,1_) in the 95% confidence interval, which was calculated in a two-way mixed effects model based on absolute agreement measures [32, 33]. An ICC value less than 0.5 indicates poor reliability, a value between 0.5 and 0.75 indicates moderate reliability, a value between 0.75 and 0.9 indicates good reliability and a value higher than 0.9 indicates excellent reliability [32]. The interval period between repeated tests is often 1 or 2 weeks [34]. The measurement errors could be assessed by the standard error of measurement (SEM) and the smallest detectable change (SDC). A score higher than the value of SDC reflects the “real” change beyond the measurement errors [34, 35].

#### Measures of validity

Content validity indicates the ability of an instrument to reflect the domain of interest and the conceptual definition of a construct [15]. Floor/ceiling effects could be used to describe the content validity. If more than 15% of the subjects reach the lowest or highest possible scores, their deterioration or improvement cannot be detected by the instrument, which means floor/ceiling effects are present [36], indicating limited content validity [34].

Construct validity indicates the ability of an instrument to measure the construct it was designed to measure [15]. In this study, construct validity was examined by exploratory factor analysis (EFA), convergent and discriminant validity and known group validity [37]. EFA was utilized to explore the factor structure by using principal component analysis with varimax rotation. Items with factor loadings lower than 0.3 have no significant correlation with any factor and should be removed [38].

If a “gold standard” is absent, convergent and discriminant validity can be assessed to verify the correlation between the assessed instrument and other existing and valid measures [37]. In this study, Spearman rank correlation analysis was utilized to calculate the correlation between the ProFitMap-neck and the NDI, the VAS, and the SF-36. A Spearman correlation value is considered small when it is between 0.1 and 0.3; moderate, between 0.3–0.5; high, ≥0.5 [39]. We hypothesized moderate negative correlations between the CHN-ProFitMap-neck and the NDI and VAS; moderate positive correlations between the CHN-ProFitMap-neck and the SF-36.

Known group validity indicates the sensitivity of an instrument between different groups [15]. If the scores of two different groups are proven significantly different (*P* < 0.05) via the independent-sample T test, it means that the instrument is able to detect the difference between different groups [37]. We hypothesized that the CHN-ProFitMap-neck scores in the CNP group would be significantly lower than those in the non-CNP group.

## Results

### Demographics

Details of demographic characteristics are presented in Table 1. Two hundred twenty patients with CNP and 100 individuals without neck pain were included. All questionnaires were valid. No significant difference was found in the age of the two groups. The CNP group included 57 males (25.91%) and 163 females (74.09%); the non-CNP group included 37 males (37%) and 63 females (63%).

**Table 1:** Baseline demographics of the participants

Variables	The CNP group (Mean ± SD) (*n* = 220)	The non-CNP group (Mean ± SD) (*n* = 100)
Age (years)	24.13 ± 5.95	24.13 ± 4.99
Gender, no. (%)		
Male	57 (25.91)	37 (37)
Female	163 (74.09)	63 (63)
Weight (kg)	59.25 ± 11.39	62.71 ± 11.88
Height (cm)	167.16 ± 7.95	169.68 ± 8.48
BMI (kg/m^2^)	21.09 ± 3.02	21.60 ± 2.53
Education, no. (%)		
Junior college	8 (3.64)	4 (4)
Bachelor	134 (60.91)	61 (61)
Master	69 (31.36)	32 (32)
PhD	9 (4.09)	3 (3)
Occupation, no. (%)		
Student	154 (70)	73 (73)
Office worker	66 (30)	27 (27)
Instrument outcomes		
VAS (0–10)	4.20 ± 1.86	–
NDI (0–50)	8.05 ± 3.79	–
SF-36 (0–100)		
BP	70.15 ± 12.99	–
PF	91.67 ± 8.52	
RP	63.70 ± 36.92	
GH	63.36 ± 17.89	
VT	62.23 ± 14.80	
SF	91.10 ± 17.48	
RE	46.52 ± 42.07	
MH	62.51 ± 14.63	
ProFitMap-neck (0–100)		
Symptom-frequency	60.77 ± 12.44	89.93 ± 6.40
Symptom-intensity	63.42 ± 11.48	90.45 ± 6.04
Functional limitation	77.98 ± 11.16	97.73 ± 2.81
Total score	66.11 ± 10.46	92.09 ± 4.96

*CNP* Chronic neck pain, *BMI* Body Mass Index, *VAS* Visual Analogue Scale, *NDI* Neck Disability Index, *SF-36* The 36-item Short Form Health Survey, *BP* Body pain, *PF* Physical functioning, *RP* Role limitations due to physical problems, *GH* General health, *VT* Vitality, *SF* Social function, *RE* Role limitations due to emotional problems, *MH* Mental health, *ProFitMap-neck* Profile Fitness Mapping questionnaire

### Translation and cross-cultural adaptation

In the translation stage, the two translators added “objects” to items 6, 7, and 8 in the functional limitation scale, which were changed into “carry objects” “lift objects” and “throw objects”, to fit in the Chinese grammar. In the stage of expert committee synthesis, item 9 in the functional limitation scale was changed to “put on and take off a T-shirt/ sweater” in consideration of experience equivalence due to the diverse climate in China. To make item 10 in the symptom scale more intelligible, the “jaw problems” was further explained to “jaw problems (pain or discomfort)” after negotiating with associate professor Martin Björklund.

In the pilot test, 15 participants with CNP were recruited to complete the CHN-ProFitMap-neck and offered their opinions on the items, questions and answers. First, the format of the questionnaire was modified. The answers were attached to each item rather than only one answer column being attached at the end of the questionnaire. Second, the phrase “Does your neck problem affect” was added to item 25 “your sleep?” and item 26 “your mood?” in the symptom scale; the phrase “What do you say about” was added to item 17 “the condition of your neck?” and item 18 “your general health?”. Third, the answers in the functional limitation scale were simplified: answer 1 “very good, no problem”; answer 2 “good, easily”; answer 3 “rather good, rather easy”; answer 4 “rather bad, rather difficult”; answer 5 “bad, difficult”; answer 6 “very bad, very difficult/ impossibly”, because the original answers were redundant.

### Reliability

#### Internal consistency

The Cronbach’s α values were 0.89 for the symptom-frequency index, 0.88 for the symptom-intensity index, 0.92 for the functional limitation scale, and 0.95 for the total score, indicating that the CHN-ProFitMap-neck had adequate internal consistency (Table 2).

**Table 2:** Internal consistency of the CHN-ProFitMap-neck

CHN-ProFitMap-neck	Cronbach’s α
Symptom-frequency	0.89
Symptom-intensity	0.88
Functional limitation	0.92
Total score	0.95

*CHN-ProFitMap-neck* Chinese version of ProFitMap-neck

#### Test-retest reliability

The ICC_3A,1_ value was 0.80 (95% CI, 0.72–0.86) for the symptom-frequency index, 0.78 (95% CI, 0.68–0.85) for the symptom-intensity index, 0.84 (95% CI, 0.77–0.89) for the functional limitation index, and 0.85 (95% CI, 0.82–0.88) for the total score (Table 3). The values of SEM and SDC were also listed in Table 3. The SEM values ranged from 4.43 to 5.29 (symptom-frequency index: 5.29, symptom-intensity index: 5.16, functional limitation index: 4.43, total score: 5.06). The SDC values ranged from 12.29 to 14.67 (symptom-frequency index: 14.67, symptom-intensity index: 14.31, functional limitation index: 12.29, total score: 14.03).

**Table 3:** Intraclass correlation coefficient of the CHN-ProFitMap-neck

	ICC (95% confidence interval) Lower-upper bound	SEM	SDC
CHN-ProFitMap-neck			
Symptom-frequency	0.80(0.72–0.86)	5.29	14.67
Symptom-intensity	0.78(0.68–0.85)	5.16	14.31
Functional limitation	0.84(0.77–0.89)	4.43	12.29
Total score	0.85(0.82–0.88)	5.06	14.03

*CHN-ProFitMap-neck* Chinese version ProFitMap-neck, *SEM* Standard error of measurement, *SDC* Smallest detectable change

### Validity

In the CNP group, the scores of the symptom-frequency index ranged from 21.38 to 89.97, the scores of the symptom-intensity index ranged from 23.09 to 90.03, the scores of the functional limitation index ranged from 50.85 to 96.61, and the total scores ranged from 36.63 to 91.69, indicating that no floor/ceiling effects were present (0% highest possible score and 0% lowest possible score).

Item 25 in the symptom-frequency index and symptom-intensity index showed similar factor loadings on factor 3 and factor 4 after one EFA. Thus, item 25 was removed and EFA was conducted again. Six factors were verified for the CHN-ProFitMap-neck symptom-frequency index (Table S1) and symptom-intensity index (Table S2). In the symptom-frequency index, item 26 had a higher loading on factor 1 (0.529), and item 23 had a higher loading on factor 4 (0.516). Based on professional knowledge and conception, however, item 26 can be classified into factor 2 (0.324), and item 23 can be classified into factor 1 (0.417). Thus, in the CHN-ProFitMap-neck symptom-frequency index: factor 1 was “pain-related symptoms and abnormal movements in the neck”, including items 1–7, and items 23–24; factor 2 was “mental health”, including items 13–15, items 19, 22, 26; factor 3 was “neuromuscular disorders”, including items 8–10; factor 4 was “balance”, including items 11–12 and item 16; factor 5 was “sensory dysfunction”, including items 17–18; and factor 6 was “respiratory and digestive disorders”, including items 20–21. The same was true for the symptom-intensity index; item 23 had a higher loading on factor 4 (0.535). Based on professional knowledge and conception, however, item 23 can be classified into factor 1 (0.368). Thus, in the CHN-ProFitMap-neck symptom-intensity index: factor 1 was “pain-related symptoms in the neck”, including items 1–3, item 5, and items 23–24; factor 2 was “mental health”, including items 13–15, items 19, 22, and 26; factor 3 was “neuromuscular, respiratory and digestive disorders”, including items 8–10, and items 20–21; factor 4 was “balance”, including items 11–12 and item 16; factor 5 was “sensory dysfunction”, including items 17–18; and factor 6 was “abnormal movements in the neck”, including item 4 and items 6–7. Four factors were verified for the CHN-ProFitMap-neck functional limitation scale after EFA once (Table S3). Factor 1 was “neck activities”, including items 37–42; factor 2 was “activities of daily living”, including items 32–36; factor 3 was “functional activities”, including items 27–31; and factor 4 was “self-evaluation”, including items 43–44.

For the convergent and discriminant validity, the Spearman correlation coefficients between the CHN-ProFitMap-neck and other questionnaires (VAS, NDI, SF-36) were shown in Table 4. The CHN-ProFitMap-neck had a small to moderate negative correlation with VAS (*r* = 0.30 for the symptom-frequency index; *r* = 0.36 for the symptom-intensity index; *r* = 0.29 for total score), but no significant correlation was present between the functional limitation index and VAS (*P* > 0.05). The CHN-ProFitMap-neck had a moderate to high negative correlation with NDI. (*r* = 0.57 for the symptom-frequency index; *r* = 0.54 for the symptom-intensity index; *r* = 0.46 for the functional limitation index; *r* = 0.60 for total score). The CHN-ProFitMap-neck symptom-frequency index had a moderate to high positive correlation with the SF-36 (*r* = 0.32–0.52). The symptom-intensity index had a small to moderate positive correlation with the SF-36 (*r* = 0.26–0.47). The functional limitation index had a small to moderate positive correlation with the SF-36 (*r* = 0.21–0.38) rather than the “mental health” domain (*P* > 0.05). The total score had a moderate positive correlation with the SF-36 (*r* = 0.33–0.49).

**Table 4:** Correlation among CHN-ProFitMap-neck and the VAS, NDI, and SF-36

		CHN-ProFitMap-neck			
		symptom-frequency	Symptom-intensity	Functional limitation	Total score
VAS		−.30^**^	−.36^**^	−.06	−.29^**^
NDI		−.58^**^	−.54^**^	−.46^**^	−.60^**^
SF-36	BP	.41^**^	.37^**^	.21^**^	.37^**^
	PF	.33^**^	.28^**^	.38^**^	.37^**^
	RP	.32^**^	.27^**^	.31^**^	.33^**^
	GH	.48^**^	.44^**^	.31^**^	.47^**^
	VT	.52^**^	.47^**^	.26^**^	.49^**^
	SF	.46^**^	.45^**^	.27^**^	.47^**^
	RE	.39^**^	.37^**^	.26^**^	.39^**^
	MH	.48^**^	.47^**^	.14	.43^**^

*CHN-ProFitMap-neck* Chinese version ProFitMap-neck, *NDI* Neck disability index, *VAS* Visual analogue scale, *SF-36* 36-item short form health survey

**P < 0.01

**Fig. 2:**
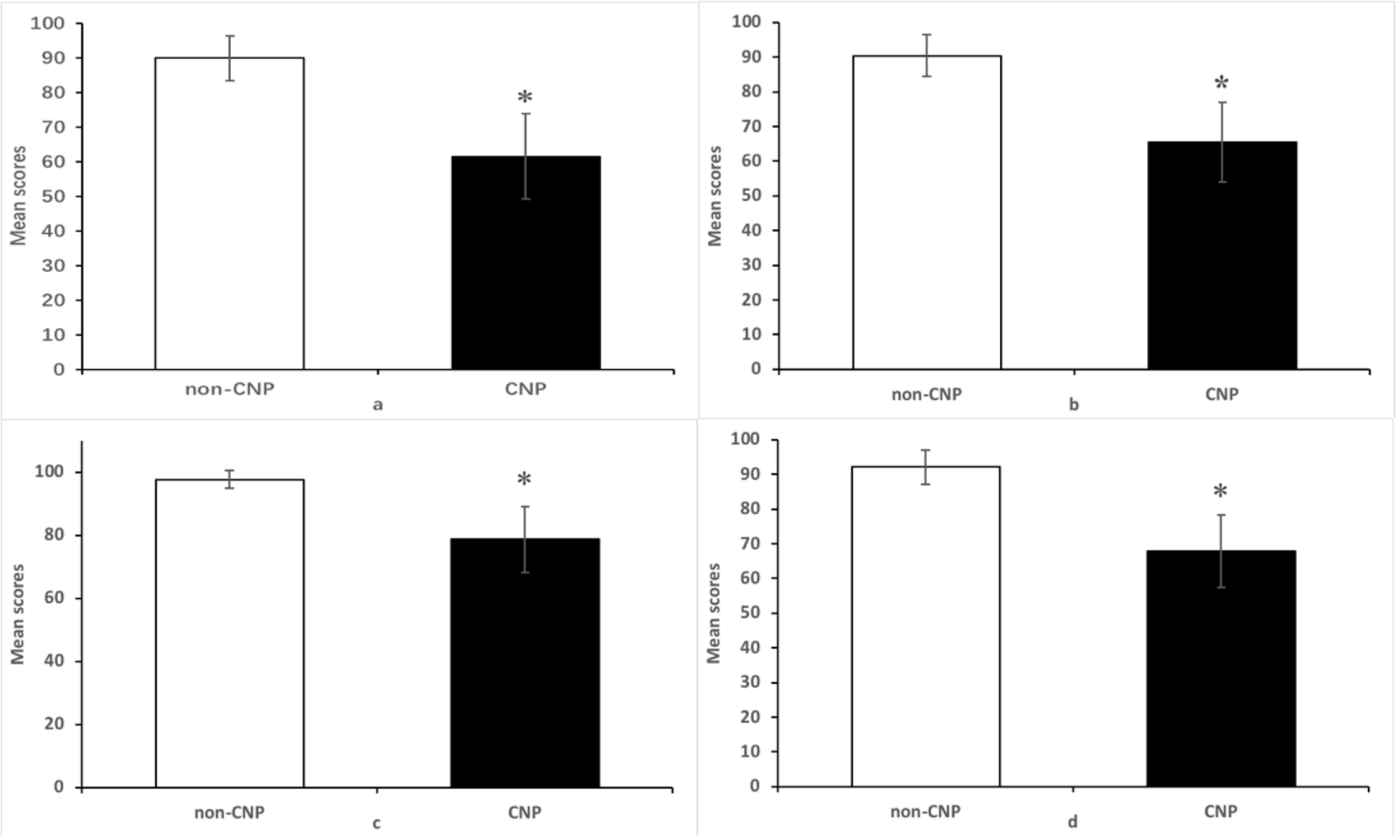
Comparison between average scores of the CNP and non-CNP. **a** symptom-frequency scale. **b** symptom-intensity scale. **c** functional limitation scale. **d** total scale. *P < 0.01

For the known group validity, 100 subjects in the CNP group were randomly chosen to be compared with 100 subjects in the non-CNP group to determine the difference in the CHN-ProFitMap-neck scores between the two groups. The scores of the symptom-frequency index, symptom-intensity index, functional limitation index, and total score were 61.62, 64.45, 78.65, and 67.80 in the CNP group and 89.93, 90.45, 97.72, 92.09 in the non-CNP group. The scores in the CNP group were significantly lower than those in the non-CNP group (*P* < 0.01) (Fig. 2).

## Discussion

The present study aimed to translate and adapt the ProFitMap-neck into the Chinese version and evaluate its psychometric properties in Chinese patients with chronic neck pain. The results of our study support that the CHN-ProFitMap-neck is a reliable and valid instrument to assess patients with chronic neck pain.

### Reliability tests

The present study showed adequate internal consistency of the CHN-ProFitMap-neck (Cronbach’s α > 0.8) [31], which was consistent with previous studies (the original ProFitMap-neck: Cronbach’s α = 0.88–0.96 [16]; the Turkish ProFitMap-neck: Cronbach’s α = 0.89–0.960 [20]; the Brazilian Portuguese ProFitMap-neck (Br-ProFitMap-neck): Cronbach’s α = 0.75–0.76 [19]).

The present study found good test-retest reliability of the CHN-ProFitMap-neck (ICC = 0.78–0.85). Good test-retest reliability was identified by ICC values (0.78–0.85), which were similar to ICC values in the Turkish study (0.73–0.84) [20] and the original ProFitMap-neck study (0.80–0.91) [16], because these studies had a similar interval period (1 week) [16, 20]. However, the ICC values were higher in the Br-ProFitMap-neck study (0.97 - 0.98) [19] because the interval period was much shorter (5 hours), allowing for better control of variables. It is worth noting that the present study chose the same ICC model (two-way mixed effects model) as the original ProFitMap-neck study [16] and the Br-ProFitMap-neck study [19], while the Turkish study did not describe the ICC model [20].

The present study showed an SEM of 4 to 5 points and an SDC of 12 to 15 points, indicating that a change of at least 12 to 15 points on CHN-ProFitMap could be regarded as the “real” change beyond the measurement errors. Our results were similar to those in the original ProFitMap-neck study (SEM = 4–6, SDC = 12–18) [16] but higher than those in the Br-ProFitMap-neck study (SEM = 2–3, SDC = 5–8) [19], which could be explained by the shorter test-retest interval period in the Br-ProFitMap-neck study. The previous study also reported that the minimal important change (MIC) of the ProFitMap-neck ranged between 6.6 and 13.6% in women with neck pain [40], while MIC was not investigated in our study.

### Validity tests

No floor/ceiling effects were present, indicating that CHN-ProFitMap-neck had good content validity. Comparable results could be observed in the original ProFitMap-neck study [16].

In previous studies, only the Br-ProFitMap-neck study performed EFA, identifying one factor (“neck symptoms”) in the symptom-frequency index, 2 factors (“balance” and “general”) in the symptom-intensity index and 2 factors (“posture and activities of daily living” and “movements and health perception”) in the functional limitation index [19]. The EFA in our study verified a similar factor structure with that in the Br-ProFitMap-neck study, but more factors were extracted in our study. This could be explained by the difference in sample size in different studies and participant characteristics in different regions. A total of 220 participants (both male and female) were included in our study while 180 females were included in the Br-ProFitMap-neck study [19]. Additionally, the VAS and NDI scores in the Br-ProFitMap-neck study were higher than those in our study [19].

The convergent and discriminant validity was supported by the results of our study. The CHN-ProFitMap-neck had a mild to moderate negative correlation with VAS (r = 0.29–0.36). The correlation values were lower in the present study than in the Turkish ProFitMap-neck study (*r* = 0.499–0.864), which could be caused by the lower pain intensity of patients in our study (VAS = 4.20 in our study; VAS = 4.61 in the Turkish study [20]). Moreover, no significant correlation was present between the functional limitation index and VAS (*P* > 0.05). This could be explained by fewer functional limitation of patients in our study (NDI = 4.77 in the present study; NDI = 13.02 in the Turkish study [20]). The present study showed a moderate to high correlation between CHN-ProFitMap-neck and NDI (*r* = 0.46–0.60). The results resembled those of previous studies (*r* = 0.66–0.78 for the original ProFitMap-neck [16]; *r* = 0.56–0.71 for the Br-ProFitMap-neck [19]; *r* = 0.612–0.710 in the Turkish ProFitMap-neck [20]). The CHN-ProFitMap-neck had a small to high positive correlation with the SF-36 (*r* = 0.21–0.52). Similarly, previous studies showed a positive correlation between the ProFitMap-neck and the SF-36 (*r* = 0.31–0.64 for the original ProFitMap-neck [16]; *r* = 0.21–0.85 for the Br-ProFitMap-neck [19]; *r* = 0.138–0.522 for the Turkish ProFitMap-neck [20]). The only exception was that no significant correlation was found between the functional limitation scale and the mental health domain of the SF-36 because the function scale in the CHN-ProFitMap-neck did not include any item related to mental health.

We confirmed our initial hypothesis that the scores of the CHN-ProFitMap-neck were significantly lower in the CNP group than in the non-CNP group (*P* < 0.01), indicating that the CHN-ProFitMap-neck could distinguish between patients with chronic neck pain and individuals without neck pain. This supports the known group validity of the CHN-ProFitMap-neck. No previous studies examined known group validity; thus, the results could not be compared with those of other studies.

## Limitation

Several limitations could be noted in the present study. First, the present study did not include any patient with acute neck pain. Second, the present study did not explore the responsiveness of the CHN-ProFitMap-neck. Thus, MIC was not accessible.

## Conclusion

The CHN-ProFitMap-neck had acceptable internal consistency, test-retest reliability, content validity and construct validity. It could be used as a reliable and valid instrument to assess patients with chronic neck pain in mainland China.

## Supplementary Information

**Additional file 1: Table S1.** CHN-ProFitMap-neck symptom-frequency index (item 25 removed). **Table S2.** CHN-ProFitMap-neck symptom-intensity index (item 25 removed). **Table S3.** CHN-ProFitMap-neck functional limitation scale.

**Additional file 2.**

## Data Availability

The datasets used and analyzed during this study are available from the corresponding author on reasonable request.

## Abbreviations

CNP: Chronic neck pain
ICC: Intraclass correlation coefficient
CHN-ProFitMap-neck: Chinese version ProFitMap-neck
NDI: Neck disability index
VAS: Visual analogue scale
SF-36: The 36-item short form health survey
PROMs: The patient-reported outcome measures
ProFitMap-neck: Profile Fitness Mapping Neck Questionnaire
ICF: International Classification of Functioning, Disability and Health
PF: Physical functioning
RP: Role limitations due to physical problems
BP: Body pain
GH: General health
VT: Vitality
SF: Social functioning
RE: Role limitations due to emotional problems
MH: Mental health
SEM: Standard error of measurement
SDC: Smallest detectable change
EFA: Exploratory factor analysis
KMO: Kaiser-Meyer-Olkin
BTS: Bartlett’s Test of Sphericity
Br-ProFitMap-neck: Brazilian Portuguese ProFitMap-neck
MIC: Minimal important change
