# Supplementary material for: Cross-cultural adaptation, reliability and validity tests of the Chinese version of the Profile Fitness Mapping neck questionnaire
Source: BMC Musculoskelet Disord. 2023 Jan 12;24:26. doi: 10.1186/s12891-022-06087-x (PMC9835234; doi:10.1186/s12891-022-06087-x)
Supplement: Supplementary file 1 — Additional file 1: Table S1. CHN-ProFitMap-neck symptom-frequency index (item 25 removed). Table S2. CHN-ProFitMap-neck symptom-intensity index (item 25 removed). Table S3. CHN-ProFitMap-neck functional limitation scale. [file 12891_2022_6087_MOESM1_ESM.docx]

Table S1 CHN-ProFitMap-neck symptom-frequency index (item 25 removed)

| item | Factor 1 | Factor 2 | Factor 3 | Factor 4 | Factor 5 | Factor 6 |
| --- | --- | --- | --- | --- | --- | --- |
| 1 | **.802** |  |  |  |  |  |
| 2 | **.782** |  |  |  |  |  |
| 5 | **.721** |  |  |  |  |  |
| 3 | **.682** |  |  |  |  |  |
| 24 | **.549** |  |  |  |  |  |
| 7 | **.547** |  |  |  |  |  |
| 26 | **.**529 | **.324** |  |  |  |  |
| 6 | **.516** |  |  |  |  |  |
| 4 | **.437** |  |  |  |  |  |
| 15 |  | **.865** |  |  |  |  |
| 22 |  | **.826** |  |  |  |  |
| 14 |  | **.632** |  |  |  |  |
| 13 |  | **.505** |  |  |  |  |
| 19 |  | **.484** |  |  |  |  |
| 9 |  |  | **.727** |  |  |  |
| 8 |  |  | **.702** |  |  |  |
| 10 |  |  | **.641** |  |  |  |
| 11 |  |  |  | **.752** |  |  |
| 12 |  |  |  | **.622** |  |  |
| 16 |  |  |  | **.535** |  |  |
| 23 | **.471** |  |  | .516 |  |  |
| 18 |  |  |  |  | **.840** |  |
| 17 |  |  |  |  | **.754** |  |
| 20 |  |  |  |  |  | **.772** |
| 21 |  |  |  |  |  | **.488** |
| **Descriptive statistics** | | | | | | |
| Eigenvalue | 3.896 | 2.986 | 2.596 | 2.223 | 1.891 | 1.470 |
| Percentage of cumulative variance | 15.585 | 27.527 | 37.912 | 46.802 | 54.368 | 60.246 |
| KMO | 0.851 | | | | | |
| BTS | χ^2^ = 2056.496/p = 0.000 | | | | | |

KMO: Kaiser-Meyer-Olkin; BTS: Bartlett’s Test of Sphericity.

Table S2 CHN-ProFitMap-neck symptom-intensity index (item 25 removed)

| item | Factor 1 | Factor 2 | Factor 3 | Factor 4 | Factor 5 | Factor 6 |
| --- | --- | --- | --- | --- | --- | --- |
| 2 | **.818** |  |  |  |  |  |
| 1 | **.801** |  |  |  |  |  |
| 3 | **.721** |  |  |  |  |  |
| 5 | **.675** |  |  |  |  |  |
| 24 | **.455** |  |  |  |  |  |
| 15 |  | **.841** |  |  |  |  |
| 22 |  | **.759** |  |  |  |  |
| 14 |  | **.660** |  |  |  |  |
| 13 |  | **.514** |  |  |  |  |
| 19 |  | **.483** |  |  |  |  |
| 26 |  | **.397** |  |  |  |  |
| 20 |  |  | **.790** |  |  |  |
| 21 |  |  | **.729** |  |  |  |
| 10 |  |  | **.465** |  |  |  |
| 8 |  |  | **.463** |  |  |  |
| 9 |  |  | **.441** |  |  |  |
| 11 |  |  |  | **.808** |  |  |
| 12 |  |  |  | **.609** |  |  |
| 16 |  |  |  | **.548** |  |  |
| 23 | **.368** |  |  | .535 |  |  |
| 18 |  |  |  |  | **.721** |  |
| 17 |  |  |  |  | **.710** |  |
| 6 |  |  |  |  |  | **.783** |
| 7 |  |  |  |  |  | **.709** |
| 4 |  |  |  |  |  | **.371** |
| **Descriptive statistics** | | | | | | |
| eigenvalue | 3.090 | 2.835 | 2.497 | 2.346 | 1.866 | 1.851 |
| Percentage of umulative varaince | 12.361 | 23.703 | 33.692 | 43.075 | 50.539 | 57.944 |
| KMO | 0.829 | | | | | |
| BTS | χ^2^ = 1879.027/p = 0.000 | | | | | |

KMO: Kaiser-Meyer-Olkin; BTS: Bartlett’s Test of Sphericity.

Table S3 CHN-ProFitMap-neck functional limitation scale

| item | Factor 1 | Factor 2 | Factor 3 | Factor 4 |
| --- | --- | --- | --- | --- |
| 40 | **.859** |  |  |  |
| 39 | **.857** |  |  |  |
| 41 | **.830** |  |  |  |
| 42 | **.829** |  |  |  |
| 38 | **.665** |  |  |  |
| 37 | **.652** |  |  |  |
| 34 |  | **.842** |  |  |
| 33 |  | **.774** |  |  |
| 35 |  | **.763** |  |  |
| 32 |  | **.742** |  |  |
| 36 |  | **.632** |  |  |
| 27 |  |  | **.851** |  |
| 28 |  |  | **.838** |  |
| 29 |  |  | **.800** |  |
| 31 |  |  | **.657** |  |
| 30 |  |  | **.560** |  |
| 43 |  |  |  | **.805** |
| 44 |  |  |  | **.728** |
| **Descriptive statistics** | | | | |
| Eigenvalue | 4.220 | 3.665 | 3.268 | 1.545 |
| Percentage of cumulative variance | 23.443 | 43.804 | 61.962 | 70.543 |
| KMO | 0.867 | | | |
| BTS | χ^2^ = 2755.347/p = 0.000 | | | |

KMO: Kaiser-Meyer-Olkin; BTS: Bartlett’s Test of Sphericity.
