# Supplementary material for: Cross-cultural adaptation, reliability and validity tests of the Chinese version of the Profile Fitness Mapping neck questionnaire
Source: BMC Musculoskelet Disord. 2023 Jan 12;24:26. doi: 10.1186/s12891-022-06087-x (PMC9835234; doi:10.1186/s12891-022-06087-x)
Supplement: Supplementary file 2 — Additional file 2. [file 12891_2022_6087_MOESM2_ESM.pdf]

Alfta Rehab Center

Profile Fitness Mapping questionnaires
ProFitMap-nacke, reviderad version

Skala för symtom

**Upplever Du:**		**Hur ofta?** Svar 1-6	**Hur mycket?** Svar 7-12
1	Stelhet i nacken?		
2	Ömhet i nacken?		
3	Spänningskänsla i nacken?		
4	Knäppningar i nacken?		
5	Trötthet i nacken?		
6	Svaghet i nacken?		
7	Låsningar i nacken?		
8	Fumlighet i händerna?		
9	Känselstörningar?		
10	Käkbesvär?		
11	Yrsel?		
12	Balanssvårigheter?		
13	Retlighet ("kort stubin")?		
14	Nedstämdhet?		
15	Stresskänslighet?		
16	Illamående?		
17	Ljuskänslighet?		
18	Ljudkänslighet?		
19	Koncentrationssvårigheter?		
20	Sväljningsbesvär?		
21	Andningsbesvär?		
22	Ångest?		
23	Smärta i nacken vid aktivitet?		
24	Värk i nacken i vila?		
**Påverkar nackbesvären:**			
25	Din sömn?		
26	Ditt humör?		

OBS!

Fyll i **båda** kolumnerna!

Hur ofta?

Svara med endera av siffrorna 1-6

1. Aldrig/mycket sällan
2. Sällan
3. Ganska sällan
4. Ganska ofta
5. Ofta
6. Mycket ofta/alltid

Hur mycket?

Svara med endera av siffrorna 7-12

7. Ingenting/ingen alls
8. Svag, lite(t)
9. Ganska svag, ganska lite
10. Ganska stark, ganska mycket
11. Stark, mycket
12. Nästan outhärdligt/ outhärdligt, allt/maximalt

Alfta Rehab Center

Profile Fitness Mapping questionnaires
ProFitMap-nacke, reviderad version

Skala för funktionsbegränsningar

**Klarar Du**, pga nackbesvären, **att:**	**Hur?** Svar 1-6
1 Stå?	
2 Gå?	
3 Sitta?	
4 Ligga?	
5 Springa?	
6 Bära?	
7 Lyfta?	
8 Kasta?	
9 Ta på och av tröja?	
10 Ta på och av sockor?	
11 Böja huvudet framåt?	
12 Böja huvudet bakåt?	
13 Böja huvudet till höger?	
14 Böja huvudet till vänster?	
15 Vrida huvudet till höger?	
16 Vrida huvudet till vänster?	

Svara med endera av siffrorna 1-6

1. Mycket bra,
   inga problem,
   mycket tillfredsställande.
   mycket sannolikt
2. Bra, lätt,
   tillfredsställande,
   sannolikt
3. Ganska bra, ganska lätt,
   ganska tillfredsställande,
   ganska sannolikt
4. Ganska dåligt, ganska svårt,
   ganska otillfredsställande,
   ganska osannolikt
5. Dåligt, svårt,
   otillfredsställande,
   osannolikt
6. Mycket dåligt,
   mycket svårt/omöjligt
   mycket otillfredsställande
   mycket osannolikt

Vad anser Du om:

17 Tillståndet i Din nacke?	
18 Ditt allmäntillstånd?	

Poängberäkning för ProFitMap-nacke.

Tabellen visar viktning och maximal poäng för varje fråga i ProFitMap-nacke, och poängberäkningen för varje index. Frekvens (f) är svaret på frågan *hur ofta* sympomen upplevs (6-poängsskala från 1 = Aldrig/mycket sällan, till 6 = Mycket ofta/alltid). Intensitet (i) är svaret på frågan *hur mycket* symtomen upplevs (6-poängsskala från Ingenting/ingen alls, till 12 = Nästan outhärdligt/outhärdligt, allt/maximalt). Svaren på frågorna i skalan för funktionsbegränsningar (fb) har en spännvidd från 1 = Mycket bra, inga problem, mycket tillfredsställande mycket sannolikt, till 6 = Mycket dåligt, mycket svårt/omöjligt, mycket otillfredsställande, mycket osannolikt.
Resultatet från varje index uttrycks som procent av maximala poängsumman, där 100% är det bästa möjliga resultatet. Justeringar för icke-besvarade frågor görs genom att dra av den maximala poängen från dessa frågor från nämnaren innan procentberäkningen.

**Skala för symtom (s)**					**Skala för funktionsbegränsningar(fb)**			
**Fråga$_s$**	**Vikt (V_s)**	**Poäng frekvens index**	**Poäng intensitet index**	**Max poäng**	**Fråga$_{fb}$**	**Vikt (V_{fb})**	**Poäng funktion index**	**Max poäng**
1	2	$(6-f_1)*V_s$	$(12-i_1)* V_s$	10	1	2,4	$(6-fb_1)*V_{fb}$	12
2	2	$(6-f_2)*V_s$	$(12-i_2)* V_s$	10	2	3	$(6-fb_2)*V_{fb}$	15
3	2	$(6-f_3)*V_s$	$(12-i_3)* V_s$	10	3	2,4	$(6-fb_3)*V_{fb}$	12
4	1	$(6-f_4)*V_s$	$(12-i_4)* V_s$	5	4	3	$(6-fb_4)*V_{fb}$	15
5	1,2	$(6-f_5)*V_s$	$(12-i_5)* V_s$	6	5	2	$(6-fb_5)*V_{fb}$	10
6	2	$(6-f_6)*V_s$	$(12-i_6)* V_s$	10	6	1,2	$(6-fb_6)*V_{fb}$	6
7	2,4	$(6-f_7)*V_s$	$(12-i_7)* V_s$	12	7	1,2	$(6-fb_7)*V_{fb}$	6
8	2,4	$(6-f_8)*V_s$	$(12-i_8)* V_s$	12	8	1,2	$(6-fb_8)*V_{fb}$	6
9	2,4	$(6-f_9)*V_s$	$(12-i_9)* V_s$	12	9	2,4	$(6-fb_9)*V_{fb}$	12
10	2,4	$(6-f_{10})*V_s$	$(12-i_{10})* V_s$	12	10	2,4	$(6-fb_{10})*V_{fb}$	12
11	3	$(6-f_{11})*V_s$	$(12-i_{11})* V_s$	15	11	3	$(6-fb_{11})*V_{fb}$	15
12	2,4	$(6-f_{12})*V_s$	$(12-i_{12})* V_s$	12	12	3	$(6-fb_{12})*V_{fb}$	15
13	2	$(6-f_{13})*V_s$	$(12-i_{13})* V_s$	10	13	3	$(6-fb_{13})*V_{fb}$	15
14	2	$(6-f_{14})*V_s$	$(12-i_{14})* V_s$	10	14	3	$(6-fb_{14})*V_{fb}$	15
15	2	$(6-f_{15})*V_s$	$(12-i_{15})* V_s$	10	15	3	$(6-fb_{15})*V_{fb}$	15
16	4	$(6-f_{16})*V_s$	$(12-i_{16})* V_s$	20	16	3	$(6-fb_{16})*V_{fb}$	15
17	1,2	$(6-f_{17})*V_s$	$(12-i_{17})* V_s$	6	17	4	$(6-fb_{18})*V_{fb}$	20
18	1,2	$(6-f_{18})*V_s$	$(12-i_{18})* V_s$	6	18	4	$(6-fb_{19})*V_{fb}$	20
19	3	$(6-f_{19})*V_s$	$(12-i_{19})* V_s$	15				
20	2	$(6-f_{20})*V_s$	$(12-i_{20})* V_s$	10				
21	2	$(6-f_{21})*V_s$	$(12-i_{21})* V_s$	10				
22	2	$(6-f_{22})*V_s$	$(12-i_{22})* V_s$	10				
23	8	$(6-f_{23})*V_s$	$(12-i_{23})* V_s$	40				
24	8	$(6-f_{24})*V_s$	$(12-i_{24})* V_s$	40				
25	3,6	$(6-f_{25})*V_s$	$(12-i_{25})* V_s$	18				
26	3,6	$(6-f_{26})*V_s$	$(12-i_{26})* V_s$	18				

Referens:
Björklund M, Hamberg J, Heiden M, Barnekow-Bergkvist M. (2012) The ProFitMap-neck - reliability and validity of a questionnaire for measuring symptoms and functional limitations in neck pain. Disability and Rehabilitation 34, 1096-1107
